# USE OF EYE TRACKING TECHNOLOGY TO DETECT MILD COGNITIVE IMPAIRMENT

**DOI:** 10.1093/geroni/igad104.0045

**Published:** 2023-12-21

**Authors:** Quinn Kennedy, Linda Chao, Dorion Liston

**Affiliations:** neuroFit, Carmel, California, United States; University of California, San Francisco, San Francisco, California, United States; neuroFit, Mountain View, California, United States

## Abstract

Early detection of dementia is key to helping individuals and their families cope. Use of eye tracking technology to measure eye movements could provide an objective and sensitive measure of cognitive impairment (Hodgson et al, 2019; Wang et al, 2020). In this pilot study, we predicted that eye tracking metrics differ between people with diagnosed MCI and those of healthy controls. Eleven veterans (≥ 55 years) being seen at the San Francisco VA Health Care System who either had a confirmed diagnosis of MCI or had subjective memory complaints and scored lower than a 26 on the MoCA participated. Their results were compared to that of a previously collected control sample (n = 41) (Liston et al, 2017). Participants completed a five-minute visual tracking test with 48 trials based on a classic step-ramp visual tracking paradigm that was administered on a PC computer with a camera and eye tracking capability. The visual tracking test yields 10 z-scored eye tracking metrics that are summarized in a single scalar summary score. Receiver operating characteristics (ROC) were computed and compared to the control sample. The summary score of the MCI group (median: -2.2) differed significantly from the healthy control sample (median: 0.0), which yielded a significant sensitivity of the test to presence or absence of MCI (ROC area = 0.94, p < .001). Although we view the results as preliminary due to the small sample size, results suggest that use of eye tracking technology may be a viable option for MCI detection.

